# Domestication reduces alternative splicing expression variations in sorghum

**DOI:** 10.1371/journal.pone.0183454

**Published:** 2017-09-08

**Authors:** Vincent Ranwez, Audrey Serra, David Pot, Nathalie Chantret

**Affiliations:** 1 Montpellier SupAgro, UMR AGAP, Montpellier, France; 2 CIRAD, UMR AGAP, Montpellier, France; 3 INRA, UMR AGAP, Montpellier, France; National Institute of Plant Genome Research, INDIA

## Abstract

Domestication is known to strongly reduce genomic diversity through population bottlenecks. The resulting loss of polymorphism has been thoroughly documented in numerous cultivated species. Here we investigate the impact of domestication on the diversity of alternative transcript expressions using RNAseq data obtained on cultivated and wild sorghum accessions (ten accessions for each pool). In that aim, we focus on genes expressing two isoforms in sorghum and estimate the ratio between expression levels of those isoforms in each accession. Noticeably, for a given gene, one isoform can either be overexpressed or underexpressed in some wild accessions, whereas in the cultivated accessions, the balance between the two isoforms of the same gene appears to be much more homogenous. Indeed, we observe in sorghum significantly more variation in isoform expression balance among wild accessions than among domesticated accessions. The possibility exists that the loss of nucleotide diversity due to domestication could affect regulatory elements, controlling transcription or degradation of these isoforms. Impact on the isoform expression balance is discussed. As far as we know, this is the first time that the impact of domestication on transcript isoform balance has been studied at the genomic scale. This could pave the way towards the identification of key domestication genes with finely tuned isoform expressions in domesticated accessions while being highly variable in their wild relatives.

## Introduction

Alternative splicing (AS) is the mechanism by which two or more processed mRNA isoforms result from the maturation of the same primary transcribed precursor mRNA molecule (pre-mRNA) [1]. One of the main steps of the pre-mRNA maturation is the splicing process, during which introns are removed from the pre-mRNA molecule, orchestrated by a whole array of *trans*-acting regulator proteins as well as *cis*-acting elements within the pre-mRNA itself. Occurring in all eukaryotes, AS has been extensively described and studied in humans [2] and other animals [3]. Through increasing diversity and complexity of transcriptomes, AS has two major outcomes: proteome diversification and regulation of gene expression. AS was suggested to be one of the possible origins of the large phenotypic differences among species which otherwise share a similar repertoire of protein-coding genes, as vertebrates do, for example [3].

AS is recognised to be a “pivotal step between transcription and translation” [4]. It has been described as varying according to organ, according to developmental stages and even according to cell type [5]. AS complex regulation is the guarantee of a consistent development for a given organism [6], several AS misregulations have been identified as causing diseases [7, 8]. Its role has been increasingly pointed out as a key factor of regulation in animals. The question of its prevalence in plants was much slower to emerge [9]. At the beginning of the last decade, AS started to be investigated in model species at the scale of the genome. The proportion of genes described as affected by AS has increased following the progress in sequencing technologies, to reach values of 48% and 61% of the intron-containing genes for recent estimations in rice [10] and *Arabidopsis thaliana* [11] respectively. Since RNAseq data is getting easier and cheaper to use, and bioinformatic tools are now available to process data and predict AS events (e.g. [12]) AS is now described, at the genomic scale, for many more species: *Brachypodium distachyon* [13], *Vitis vinifera* [14], *Hordeum vulgare* [15], tomato [16] and sorghum [17] to cite only a few of them. Some comparative analyses of AS have now started to be carried out on several species [18].

Regardless of the studied organism, the proportion of mRNA isoforms identified that are actually translated into functional proteins is not precisely known [19] and AS impact on plant proteome diversification is still being debated [20]. However, owing to the diversity and complexity of mRNA molecules AS generates, it is believed to play an essential role in the regulation of expression, and/or to affect translation probability, via the nonsense-mediated decay (NMD). NMD is a process during which alternatively spliced isoforms possessing a premature stop codon are degraded [21, 22]. Indeed AS induced regulation is very sensitive to environmental conditions. It has been shown that important changes in AS patterns occur in plants in response to environmental stresses (recently reviewed in [23, 24, 25]). A steady stream of new papers continuously brings additional examples of the role of AS in mechanisms involved in stress responses [26, 27]. Finally AS has been shown to play a role in plant immunity, through plant disease resistance genes (R-genes) AS (reviewed in [28]).

Although AS plays a key role in several biological processes, the question of its intraspecific variability has been raised only recently and only a few cases of plant intraspecific variability have been studied so far. In a recent study, Potenza et al. explored the AS landscape in ten grapevine cultivars [14]. They found that the AS isoforms are well conserved across individuals with up to 21% of them conserved across the 10 genotypes despite the fact that in most cases (~70%) one isoform is expressed at least ten times less strongly than the canonical forms. An open question remains concerning AS isoform repertoire variation among cultivars possibly due to variability in the splicing sites or possibly to the fine tuning of the spliceosome machinery (other regulatory elements, *cis* or *trans*), or both.

Up to now, how AS is finely tuned in a given individual, organ, or developmental step, is not known but the mere fact that AS varies according to genotypes and environmental changes [24] is a clue to its potential role in genetic adaptation. Consequently, one could wonder whether crop and animal domestication has a significant impact on the pattern of variability of AS.

All the traits making the crop different from its wild relative are grouped under the term of ‘domestication syndrome’. In the case of plants, this includes changes in secondary metabolites, modifications of plant architecture, increases in fruit size, loss of seed dormancy and alteration of dispersion capacity, to cite only the main changes. However, it is quite variable according to species, and in particular, annual crops, such as sorghum, have been shown to exhibit significantly stronger domestication syndrome than perennial ones [29]. From a genetic standpoint, domestication is a combination of genetic drift effects caused by founder sampling (the strength of the resulting bottleneck varies according to species), and of selective effects caused by the deliberate selection of alleles for the advantage they confer for human uses [30]. One of the recurrent objectives is to identify the underlying genetic architecture of adaptation and ultimately the genes controlling physiological and morphological traits for which changes are observed between crops and their wild relatives [31]. The search for such genetic/phenotypic relationships is routinely done using Quantitative Trait Locus (QTL) mapping, genome wide association study (GWAS) or selection scan approaches, although the latest do not directly explore the statistical links between allelic and phenotypic diversity.

Finally, beyond the methods aiming to correlate genetic to phenotypic variations caused by domestication, recent studies have focused on intermediate steps lying between genetic and phenotype, gene expression, in particular. Expression of 18,242 genes was surveyed in maize and teosinte, its wild ancestor [32, 33]. Changes in expression levels were observed for 600 of them, but at the genome-wide scale, the coefficient of variation of expression among lines was not significantly different in maize and teosinte [33]. When considering the subset of ‘candidate genes’ located in regions that they identify as undergoing either domestication or posterior selection, they observed a reduced variation in expression levels in maize *versus* teosinte. This could suggest that *cis*-acting regulatory regions were affected by domestication [32]. In cotton, comparative gene expression showed a parallel up-regulation of several genes of the same gene family in independently domesticated cotton species [34]. In tomato, comparative transcriptomics revealed expression divergence between cultivated and wild accessions, and a correlation between network rewiring and light responsiveness in domesticated tomato [35]. In common bean a very clear decrease of gene expression variability (18%) was also detected in domesticated beans as compared to their wild counterparts [36]. Another strategy is to focus on the transcriptome of organs which underwent major morphological changes during domestication such as glumes in wheat [37] for which decreased expression levels of genes involved in cell walls, lignin, pectins and wax biosynthesis potentially contribute to the divergence of the glume’s properties between wild and cultivated wheat. In cotton, it was shown that domestication affected the expression of many genes in fiber cells, with twice as many genes differentially expressed in fiber cell development in domesticated cotton versus wild [38]. This approach may help to understand the biological mechanisms underlying the complex links between genotype and phenotype, even if the causal mutation(s) controlling the difference of expression is (are) not identified. Additionally, as gene expression is an ‘intermediate’ trait, its analysis may help to identify genes that would have been missed through exclusive final phenotype variability analysis due to a lack of statistical power. Finally, a recent study identified a subset of genes expressing more isoforms in maize than in teosinte (wild relative of maize) but found no significant difference between their AS isoform repertoires (i.e. type of alternative splicing events: intron retention, alternative acceptor site and so on) [39]. However, whether domestication has impacted alternative splicing expression variability, and how, has not been described up to now.

In this paper, we study the impact of sorghum domestication on alternative splicing by identifying whether differential patterns of isoform expression are observed when comparing cultivated and wild compartments. Sorghum currently ranks fifth for grain production tonnage, providing staple food for 500 million people worldwide [40]. Its success is mainly due to its high level of drought tolerance and to its adaptation to a large spectrum of environmental conditions and uses. The recent release of its genome sequence [41], its phylogenetic proximity with several important C4 species (maize, switchgrass, sugarcane) and its low genome complexity contribute to its interest on a more fundamental level.

The *Sorghum bicolor* species includes three sub-species: ssp. *bicolor* (the domesticated form), ssp. *verticilliflorum* (the closest wild relative) and ssp. *drumondii* (the weedy form which corresponds to stable hybrids between the wild relatives and the cultivated types). The wild and domesticated pools are inter-fertile and intense gene flows occur (e.g. [42–45]). However a clear domestication syndrome is visible between the wild and cultivated pools. A key phenotypic difference between the cultivated and wild sorghum forms, controlled by the *SH1* gene [46], is that the cultivated type has large non shattering seeds whereas the wild type has small shattering seeds. Other traits corresponding to plant architecture (tillering), seed weight etc. are also highly divergent between these pools.

Concerning the mating system, the cultivated form does less outcrossing than the wild one, but even if selfing is predominant, outcrossing can reach up to 20% in some cultivated races such as the Guinea [47].

According to Hamblin [48], the domestication history of sorghum is complex and cannot be summarized by a single bottleneck event. Such a simple model simply does not fit their data and more complex scenario, e.g. including multiple domestications or introgression from wild congeners, have to be considered. There is, however, no doubt that sorghum domestication has induced a significant reduction of its molecular diversity. Considering a sample that is representative of the extensive diversity of sorghum together with a whole genome sequencing approach, [49] showed that nucleotide diversity estimated through ϴ_π_ and ϴ_w_ were respectively 35% and 28% lower in sorghum landraces compared to the wild genotypes. These reductions reach respectively 39% when considering the whole genome and 34% when considering the genic regions only. The present paper aims at studying whether or not this documented loss of allelic diversity is accompanied by a loss of diversity in gene isoform relative expression.

The growing evidence of widespread intraspecific variability of AS, along with its potential role in adaptation makes it susceptible to demographic and selective events. As plant domestication is a well-studied evolutionary process, during which demographic and selective effects are combined, we ask if, and how, domestication may have impacted AS. We ask also whether an extreme difference of AS patterns, between wild and cultivated accessions for a given gene, could be the signature of a selective effect on this gene AS pattern itself. Taking advantage of an mRNA dataset produced to document the domestication of several agronomical species [50] we chose to focus on sorghum for the quality of its genome assembly and annotation. To supplement [50] and [51] we used an additional sorghum accession (WS7) to be able to balance the number of accessions so that we had ten for each compartment. RNAseq data from these ten cultivated and wild sorghum accessions were mapped on the sorghum reference genome. We focused on genes for which exactly two isoforms were identified and we studied the variability of the expression ratio between those isoforms across compartments.

## Material and methods

### Sorghum genome and annotation

We used the sorghum genome assembly Sbi1.4 and the corresponding transcript annotations provided on the plantGDB database (http://www.plantgdb.org/XGDB/phplib/download.php?GDB=Sb). The gene ontology annotations of those annotated sorghum genes have been downloaded thanks to the biomart facilities of the plant ensEMBL database.

### Biological material

Ten accessions of cultivated sorghum have been used to produce the sequence information, *Sorghum bicolor* subsp. *bicolor* (denoted CS1 to CS10), and ten wild relatives (denoted WS1 to WS10), chosen in order to best represent the genetic diversity of each compartment (Table 1). Note that below we used indifferently the terms ‘population’ and ‘compartment’.

**Table 1:** Accession names and origins of sequenced sorghum accessions.

*Sorghum bicolor bicolor* (Cultivated sorghum: CS)			*Sorghum bicolor verticilliflorum* (Wild type sorghum: WS)		
Study code	Accession	Country	Study code	Accession	Country
**CS1**	SSM1049	Senegal	**WS1**	IS14564	Sudan
**CS2**	IS29876	Swaziland	**WS2**	IS18821	Egypt
**CS3**	IS30436	China	**WS3**	IS18909	Chad
**CS4**	SSM1123	Niger	**WS4**	IS18824	Ivory Coast
**CS5**	IS6193	India	**WS5**	IS18833	Malawi
**CS6**	SSM973	Senegal	**WS6**	IS14312	South Africa
**CS7**	IS14317	Swaziland	**WS7**	IS14357	Malawi
**CS8**	IS29407	Lesotho	WS8*	IS14719*	Ethiopia
**CS9**	SSM1057	Senegal	**WS9**	IS18804	USA
**CS10**	IS26554	Benin	**WS10**	IS18812	Egypt

* This accession was mis-assigned to the wild compartment (see next paragraph in M&M section).

We were mainly interested in comparing features observed within the compartment of 10 cultivated sorghum accessions, denoted as popCS_10_ below, with those observed in the sample of 10 wild sorghum accessions, denoted as popWS_10_.

Preliminary genomic analysis raised doubts concerning the assignation of the accession WS8 as a wild type. Indeed, SSR verifications and phenotypic observations of the seed lot received from the genebank revealed a misidentification. Additionally, a surprisingly low percentage of reads from accessions WS1, WS2 and WS5 could be properly mapped on the reference sorghum genome (details in result section). Thus, we removed those 4 accessions from our initial wild type sample popWS_10_ (thereby generating a sample we noted popWS_6_) and, to check for potential bias induced by sample sizes, we randomly subsampled 6 accessions in the cultivated sample. Four such subsamples were obtained (called popCS_6_1_, popCS_6_2_, popCS_6_3_, popCS_6_4_below).

We use popCS_x_ (respectively popWS_x_) to designate one of the above mentioned samples of cultivated sorghum (respectively wild sorghum) in assertions that hold for all of cultivated (respectively wild type) samples. Finally, we use popS_x_ to designate any of those sorghum samples.

### RNA extraction and sequencing

The RNAseq data used were obtained from a larger project dedicated to the comparison of cultivated plants with their wild relatives (http://www.arcad-project.org/projects/comparative-population-genomics). Tissue samples were collected from different organs, including leaves, grains, and inflorescence. Details for RNA extraction, Illumina libraries production and sequencing conditions are available in the Materials and Methods section of [50]. The cDNA libraries that contain a mixture of 65% RNA from the inflorescence, 15% from leaves and 20% from maturing seeds, for each accession, were sequenced using the Illumina mRNA-Seq, paired-end protocol on a HiSeq2000 sequencer (one run for each compartment). The paired-end reads, in the illumina FASTQ format, were cleaned using cutAdapt [52] to trim read ends of poor quality (q score below 20) and to keep only those with an average quality above 30 and a minimum length of 25 base pairs. Those data are freely available on the NCBI RSA database (Sequence Read Archive) (cultivated: SAMN05277472 to SAMN05277481; wild: SAMN06052464 to SAMN06052472 and SAMN07313361).

### Estimation of alternative transcript expression levels

Transcript expression levels have been estimated thanks to the Tuxedo pipeline [12]. This pipeline proceeds as follows. Firstly, for each accession, RNAseq reads are mapped on the reference genome using Tophat v2.0.13 [53] with bowtie2 v2.2.5 [54]. Secondly, the resulting mappings are used to enrich the initial gene and transcript predictions used, thanks to cuffmerge and cufflink, two programs of the cufflink suite v2.2.1 [55]. Finally, reads mappings and enriched annotations are combined to estimate, for each gene and accession, the expression level of every alternative transcript using cuffdiff, another program from the cufflink suite. The expression level is measured by cufflink as an ‘FPKM’ (Fragments Per Kilobase Of Exon Per Million Fragments Mapped), to account for heterogeneity of i) total number of reads per individual and ii) mRNA length.

When the average depth coverage of a gene was smaller than 5 for an accession, we considered that the corresponding expression level could not be reliably estimated and we replaced the cuffdiff estimation by a missing data (NA) for the corresponding gene in the considered accession.

### Estimation of alternative transcript expression ratios

We compare two panels of genotypes, popWS_x_ and popCS_x_, based on a subset of genes selected according to the following characteristics: i) genes expressing exactly two alternative transcripts (6,226 genes taken from the 33,795) ii) genes having an average depth coverage of at least 5 reads for every accession of popWS_x_ and popCS_x_ (*i*.*e*. no missing data) and iii) transcripts of genes both being expressed in at least one accession of popWS_x_ and at least one accession of popCS_x_. These filters, being quite stringent, still allow us to rely on more than a thousand genes for comparing any pair of wild/cultivated samples (cf. Results section). For such genes with exactly two isoforms, the alternative transcript expression levels can be summarized by a single expression ratio, denoted as e_T_-ratio below. The e_T_-ratio is simply the expression of one transcript divided by the overall expression of the gene. For a given gene, if we denote by x its e_T_-ratio then using the alternative transcript at the numerator would have led to an e_T_-ratio of 1-x. As long as the same isoform is used to calculate the e_T_-ratio for all accessions (within the cultivated and wild samples), using one isoform or the other at the numerator of a gene e_T_-ratio does not matter when comparing their diversity in cultivated versus wild type samples. To homogenize the presentation of the results among genes we therefore systematically used, for the e_T_-ratio numerator of a gene, the isoform leading to the highest average e_T_-ratio along popWS_x_ U popCS_x_, so that most of our e_T_-ratios range between 0.5 and 1 instead of being evenly spread between 0 and 1.

### Estimation of transcript expression diversity within population

For a given gene G and sample popS_x_, the diversity of the transcript expression is simply the diversity of its e_T_-ratios among the considered sample. If all e_T_-ratios of the given sample are close to 1, the ‘first’ transcript of G (*i*.*e*. the isoform which, on average, is the most expressed and hence used as the numerator of the e_T_-ratio) is much more expressed than its alternative transcript in all accessions of this population. Note that e_T_-ratios can be roughly constant among accessions of popS_x_ no matter the value of this constant. The diversity of the expression balance between the two isoforms of gene G among popS_x_ can be measured by the spread of its e_T_-ratios, which can be quantified using either their variance (denoted as σ_r_) or their inter-quartile range (denoted as iq_r_). Both measures capture the variability of the e_T_-ratios but the variance is much more sensitive to outlier e_T_-ratio values than the inter-quartile range. Similarly, we will summarize the e_T_-ratios of a gene G among the popS_x_ using either the average (denoted as avg_r_) or the median (med_r_) of the e_T_-ratios of G in popS_x_.

## Results

### Dataset characteristics

**Fig 1:**
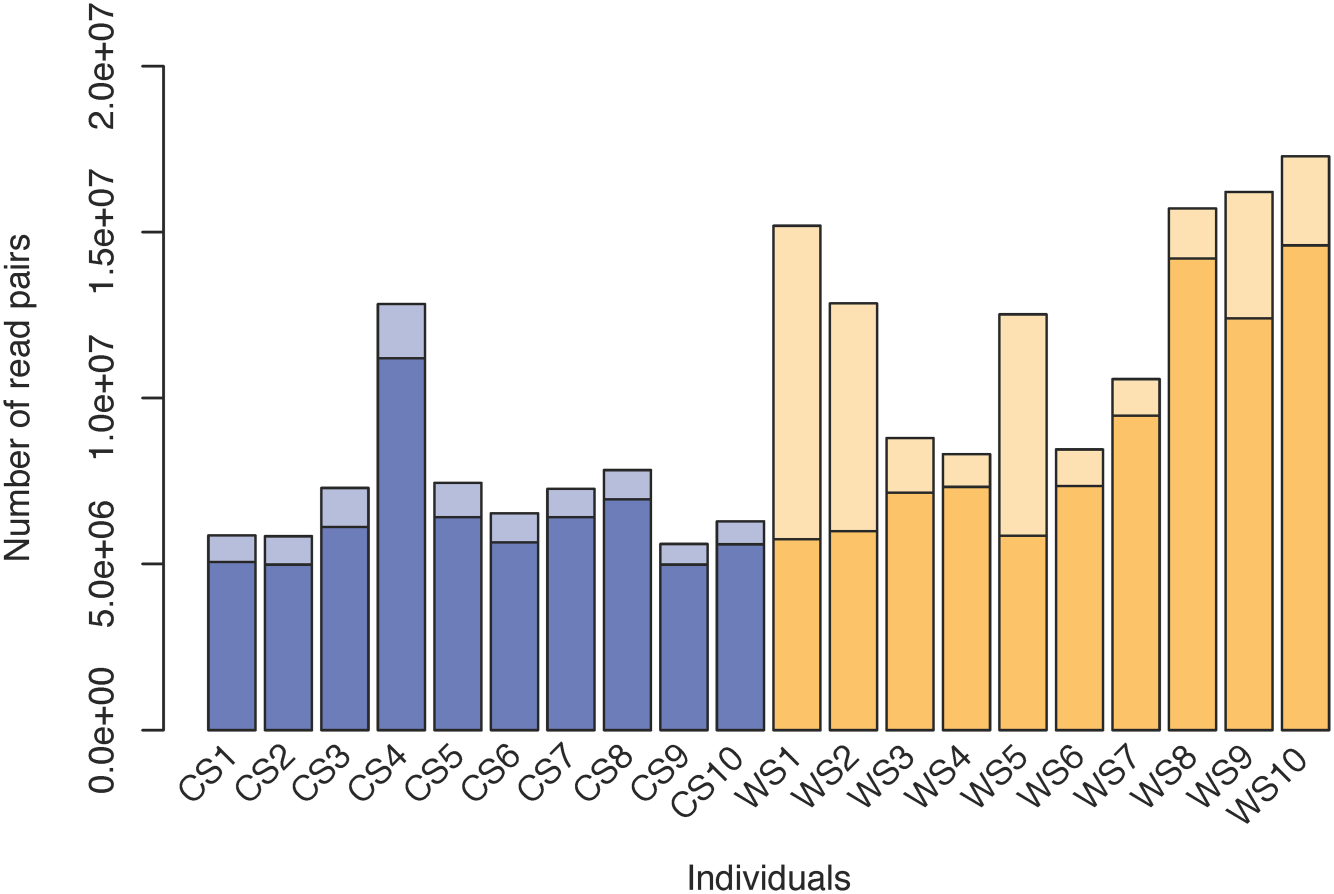
Number of clean pairs of reads mapped on the sorghum genome. The number of clean read pairs of each individual is indicated by a blue bar for cultivated sorghum accessions or an orange bar for wild sorghum accessions. For any given accession, the darker or lighter part of each bar corresponds to mapped or not mapped read pairs on the sorghum genome.

For each accession, the proportion of clean paired-end reads that successfully mapped on the sorghum V1.4 genome is provided in Fig 1. Less than 50% of the clean reads of individuals WS1 (37.8%), WS2 (46.6%) and WS5 (46.7%) have been successively mapped on the sorghum genome. This low percentage strongly contrasts with other accessions for which at least 81.3% (for individual WS3) of the read pairs have been successively mapped. Similar results were obtained with other mapping tools, showing that this is not just an artifact of the chosen mapping method. We did not find any satisfactory explanation to this low percentage of read mapping and preferred to discard those three accessions for the current analysis together with individual WS8 for which we have some suspicions of misidentification.

The impact of the gene filtering applied to our dataset, in order to base our population comparisons solely on genes with a relatively high sequencing coverage and no missing data in the compared populations, is detailed in Table 2. Note that despite quite a drastic filtering procedure, all pairwise population comparisons are conducted on more than one thousand genes.

**Table 2:** Number of genes considered for the analysis after filtering on quality and coverage.

*Compared Population*	Number of genes with 2 isoforms	and a gene coverage above 5 for each individual	and both isoforms expressed in both populations
popCS_10_ vs popWS_10_	6,226	1,385	1,134
popCS_10_ vs popWS_6_	6,226	1,635	1,358
popCS_6_1_ vs popWS_6_	6,226	1,653	1,350
popCS_6_2_ vs popWS_6_	6,226	1,698	1,397
popCS_6_3_ vs popWS_6_	6,226	1,668	1,356
popCS_6_4_ vs popWS_6_	6,226	1,682	1,383

Among the 1397 genes harboring exactly two isoforms (comparison popCS6_2 vs popWS6 Table 2, the highest number of genes among all the comparisons), 826 genes were already identified with two isoforms of transcripts in the publically available annotation, 556 genes were annotated with only one isoform of transcript, and 15 genes correspond to loci where no genes were identified. The information related to these genes is available in S1 Table (gene id, protein sequence when predictable, its length) and includes the nucleotide identifier number for mRNA sequences available in S1 File.

### Distribution of e_T_-ratio within cultivated and wild type sorghum

**Fig 2:**
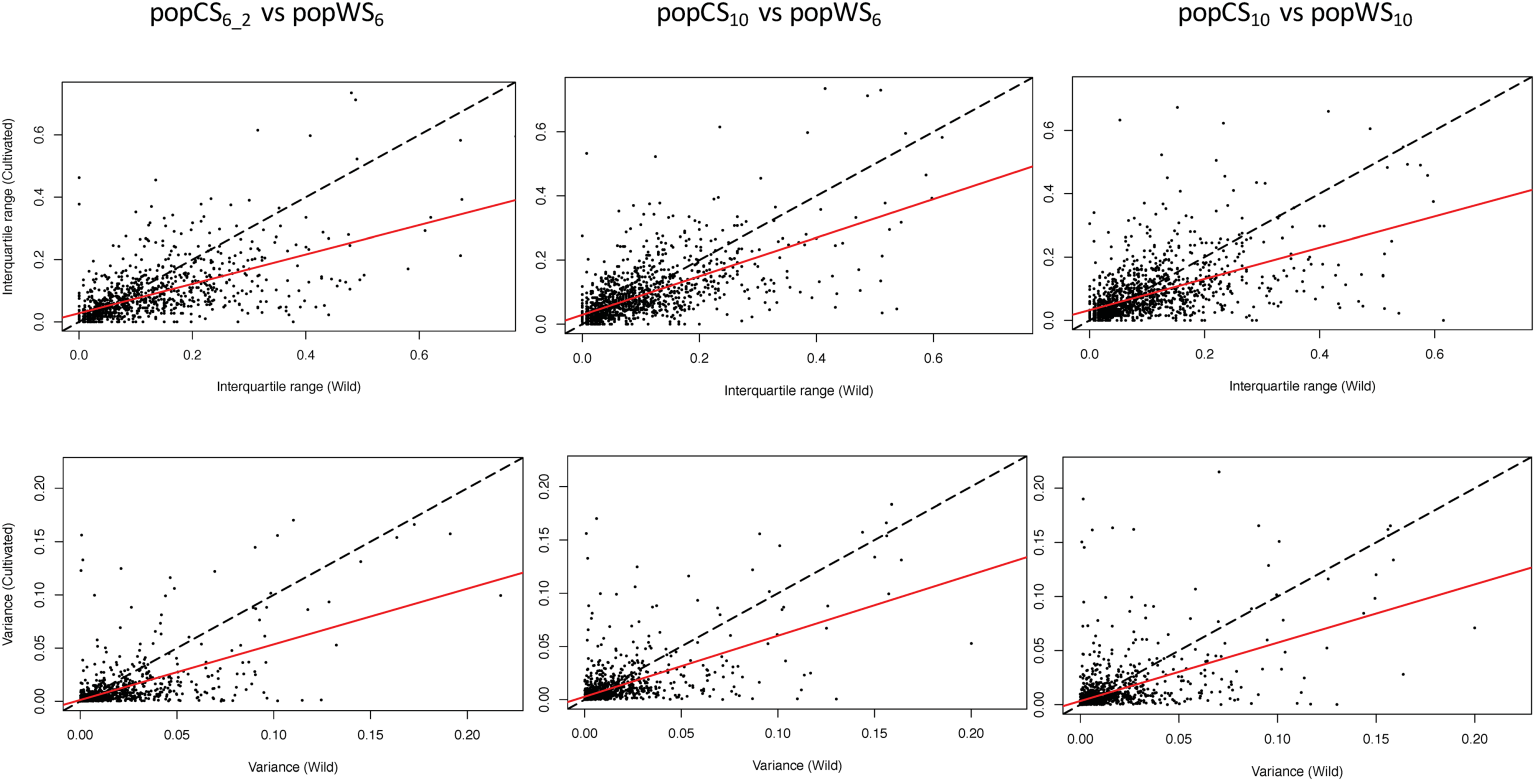
Dot plot comparison of the e_T_-ratio spread among cultivated and wild type populations. In each plot a dot represents a gene whose position corresponds to its e_**T**_-ratio spread measure by interquartile range (resp. variance) in the three top (resp. bottom) plots, observed in a sample of cultivated sorghum (abscise) and in a sample of wild sorghum accessions (ordinate). The red lines represent the linear interpolation of those points (the line slopes are provided in Tables 3 and 4) and the dashed lines depict the y = x line to ease picture interpretation.

For each pairwise population comparison, we used either e_T_-ratio mean values and variances within each population (Table 3), or e_T_-ratio medians and interquartiles (Table 4). For all popCS_x_ vs popWS_x_ comparisons, diversity of e_T_-ratios is significantly higher in cultivated populations than in domesticated ones. Indeed, most genes have an e_T_-ratio variance higher in the wild population than in the cultivated one. For instance, e_T_-ratio variance is higher in popWS_10_ than in popCS_10_ for 773 genes out of 1134 (~68%). The percentage of genes having an e_T_-ratio which is more variable in the wild population than in the cultivated population varies depending on the compared populations but is always significantly higher than 50% according to paired student t-test (highest p-value 1.63e^-110^) and Wilcoxon test (highest p-value 5.09e^-10^). The same observation holds true for comparisons based on e_T_-ratio medians and inter-quartile ranges. In all population comparisons but one, the inter-quartile range is very significantly lower in the cultivated population (p-value<1.50e^-8^ for student test and <1.79e^-9^ for Wilcoxon test). The sole minor exception is for the comparison of popCS_10_ and popWS_6_, two populations of different sizes, that do have significantly different e_T_-ratio inter-quartile ranges but with not so low p-values (p-value 0.0039 for the paired student t-test and 0.0452 for the Wilcoxon test). The simple e_T_-ratio dot plot displayed in Fig 2 gives visual prominence to this general trend of higher variance (or interquartile range) of e_T_-ratio in wild populations than in cultivated ones.

**Table 3:** Comparison of the e_T_-ratios variance between cultivated and wild sorghum samples.

	popCS_10_ popWS_10_	popCS_10_ popWS_6_	popCS_6_1_ popWS_6_	popCS_6_2_ popWS_6_	popCS_6_3_popWS_6_	popCS_6_4_popWS_6_
# σ_r_ (CS) > σ_r_ (WS)	347	599	545	592	548	532
# σ_r_ (CS) = σ_r_ (WS)	14	1	14	19	24	15
# σ_r_ (CS) < σ_r_ (WS)	773	758	791	786	784	836
slope of linear regression of (σ_r_ (CS), σ_r_ (WS))	0.5228	0.5730	0.6326	0.5393	0.5871	0.5609
Paired t-student						
mean(σ_r_ (CS) - σ_r_ (WS))	0.0058	0.0021	0.0020	0.0021	0.0020	0.0028
p-value of t-student	3.63e-^119^	4.46e^-142^	2.40e^-120^	1.63e^-110^	1.23e^-111^	7.80e^-112^
p-value Wilcoxon test	1.48e^-52^	5.09e^-10^	4.14e^-14^	6.38e^-13^	2.14e^-14^	5.99e^-22^

Each column corresponds to the comparison between a sample of cultivated genotypes and a sample of wild genotypes. In the first (resp. second and third) line are reported the number of genes with an e_T_-ratio variance (σ_r_) in the cultivated panel higher than (resp. equal to, lower than) in the wild sample. The fourth line indicates the slope of the linear interpolation of the points having σ_r_ (CS) as abscises and σ_r_ (WS) as ordinate. The mean value of the differences between σ_r_ (CS) and σ_r_ (WS) is provided in the next line, and the last two lines provide respectively the p-value of the paired t-test and the p-value of the Wilcoxon test to statistically asses the significance of the difference between σ_r_ (CS) and σ_r_ (WS) distributions.

**Table 4:** Comparison of the e_T_-ratio inter-quartile range between cultivated and wild sorghum samples. (see detailed legend in Table 3).

	popCS_10_ popWS_10_	popCS_10_ popWS_6_	popCS_6_1_ popWS_6_	popCS_6_2_ popWS_6_	popCS_6_3_popWS_6_	popCS_6_4_popWS_6_
# iq_r_ (CS) > iq_r_ (WS)	379	635	545	569	540	550
# iq_r_ (CS) = iq_r_ (WS)	80	60	59	67	63	57
# iq_r_ (CS) < iq_r_ (WS)	675	663	746	761	753	776
slope of linear regression of (iq_r_ (CS), iq_r_ (WS))	0.4714	0.6013	0.5402	0.4931	0.5058	0.4829
Paired t-student						
mean(iq_r_ (CS)—iq_r_ (WS))	0.0284	0.0060	0.0140	0.0132	0.0158	0.0175
p-value of t-student	7.44e^-25^	0.0039	1.27e^-9^	1.50e^-8^	4.09e^-12^	1.67e^-13^
p-value Wilcoxon test	6.24e^-29^	0.0452	9.25e^-11^	1.79e^-9^	2.05e^-12^	4.31e^-15^

Each column corresponds to the comparison between a sample of cultivated genotypes and a sample of wild genotypes. In the first (resp. second and third) line are reported the number of genes with an e_T_-ratio inter-quartile range (iq_r_) in the cultivated panel higher than (resp. equal to, lower than) in the wild sample. The fourth line indicates the slope of the linear interpolation of the points having iq_r_ (CS) as abscises and iq_r_ (WS) as ordinate. The mean value of the differences between iq_r_ (CS) and iq_r_ (WS) is provided in the next line, and the last two lines provide respectively the p-value of the paired t-test and the p-value of the Wilcoxon test to statistically asses the significance of the difference between iq_r_ (CS) and iq_r_ (WS) distributions.

### Organization of sorghum accessions based on their e_T_-ratios

**Fig 3:**
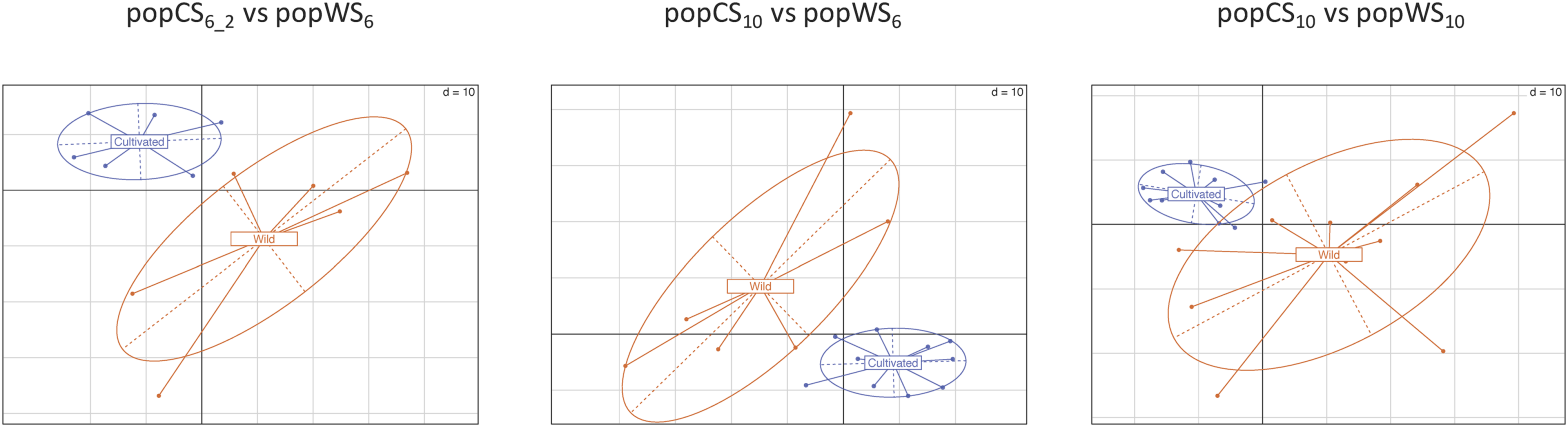
Cultivated and wild type sorghum sample 2D projection using a PCA of their e_T_-ratios. Each sorghum accession, associated with e_T_-ratios, can be seen as a point in a high dimensional space. This figure displays the projection of these points on the two first PCA axes using orange /blue dots to represent wild /cultivated individuals. The two first axes explain more than 30% of the original variability in all three cases.

The e_T_-ratios are not only less variable in the domesticated compartments, they also seem to be sufficient to correctly differentiate cultivated accessions from wild type accessions. Considering the e_T_-ratio of each gene as a coordinate, each accession can be positioned in a highly multidimensional space. The usual Principal Component Analysis (PCA) can then be used to project these accessions/points in a lower dimensional space while preserving most of the original variance. The projection obtained on the two first axis of the PCA analysis are provided in Fig 3 where cultivated accessions group together in a much more compact group than the wild individuals. Note also that, in the three PCA projections displayed in Fig 3, the two axes used for the projection explain more than 30% of the original variance.

### Genes with contrasted e_T_-ratios distribution in wild and cultivated sorghum

**Fig 4:**
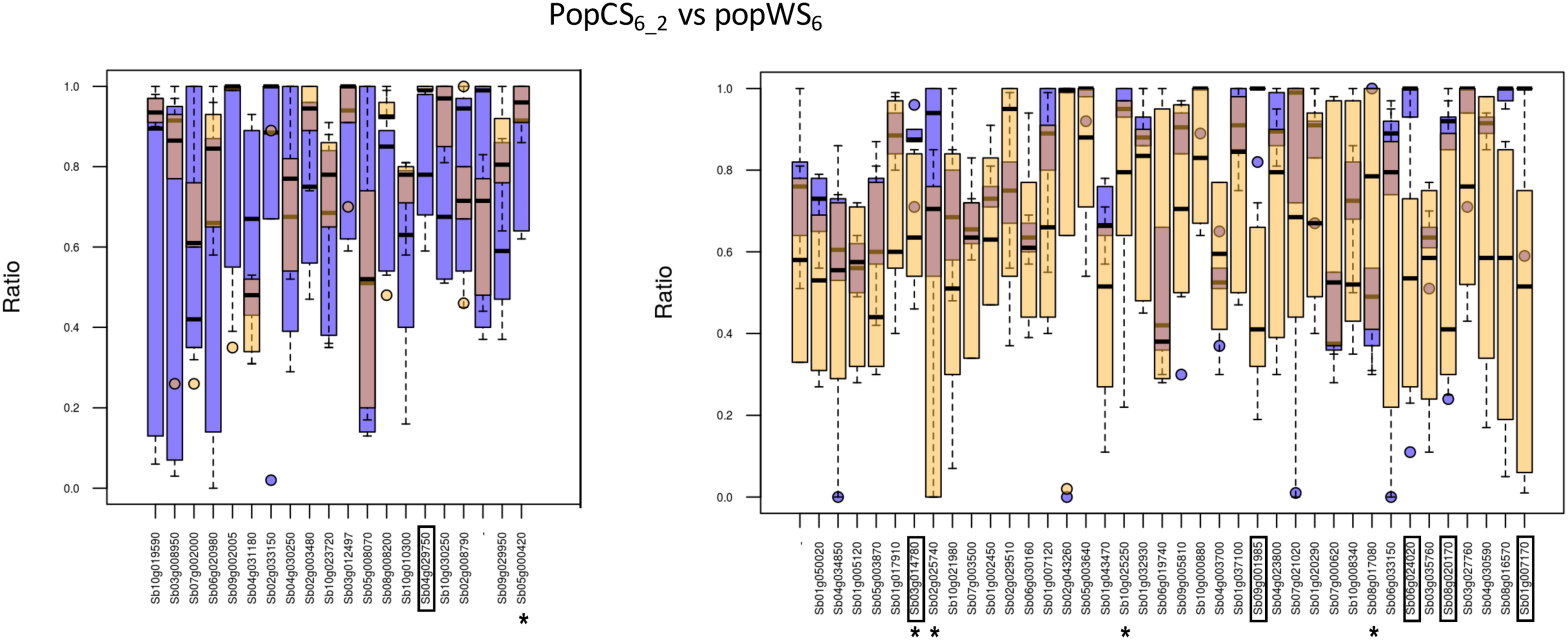
Genes with a contrasted e_T_-ratio interquartile in cultivated (popCS_6_2_) and wild (popWS_6_) samples. Box plot representations of the e_T_-ratio in the cultivated (blue) and wild (orange) samples, for genes with an e_T_-ratio more variable in cultivated (left) or wild (right) samples. The genes marked by a star are annotated by the GO term ‘regulation of biological quality’. The framed genes are common to Fig 5.

Genes with contrasted e_T_-ratio variability between the cultivated and wild compartments are potentially related to the domestication syndrome. To identify such genes, we were looking for genes having an interquartile range which differs between both populations by at least 0.2, *i*.*e*. genes such as |iq_r_ (CS)—iq_r_ (WS)| > 0.2. We found about twice as many genes with a higher interquartile range in wild compartments compared to the cultivated ones, than the opposite way around (Fig 4). All identified genes are interesting as such contrasts of e_T_-ratio, whatever their orientation, may reveal genes that have been affected by domestication.

A total of 59 genes were identified when comparing popCS_6_2_ and popWS_6_ (Fig 4), among which nineteen are consistently recovered by the three population comparisons we focus on. Twelve out of these nineteen genes are annotated by specific GO terms. To find out if some GO terms are over represented in this set of 12 genes with respect to the set of 921 annotated genes common to the three population comparisons, we relied on the AgriGO webserver (http://bioinfo.cau.edu.cn/agriGO/analysis.php). This enrichment analysis was done using Singular Enrichment Analysis (SEA) with hypergeometric test, p-value threshold at 0.05 and Bonferroni correction for multiple testing. A single annotation is found to be over-represented by this test (p-value 0.00042), the GO term ‘regulation of biological quality’ (GO:0065008). This GO term has a frequency of 0.42 (5/12) in our subset versus a frequency of 0.04 (40/921) in the subset of annotated genes common to the 3 population comparisons. The five genes annotated by this GO term are Sb02g025740, Sb03g014780, Sb05g000420, Sb08g017080, and Sb10g025250 (marked by a star in Fig 4).

**Fig 5:**
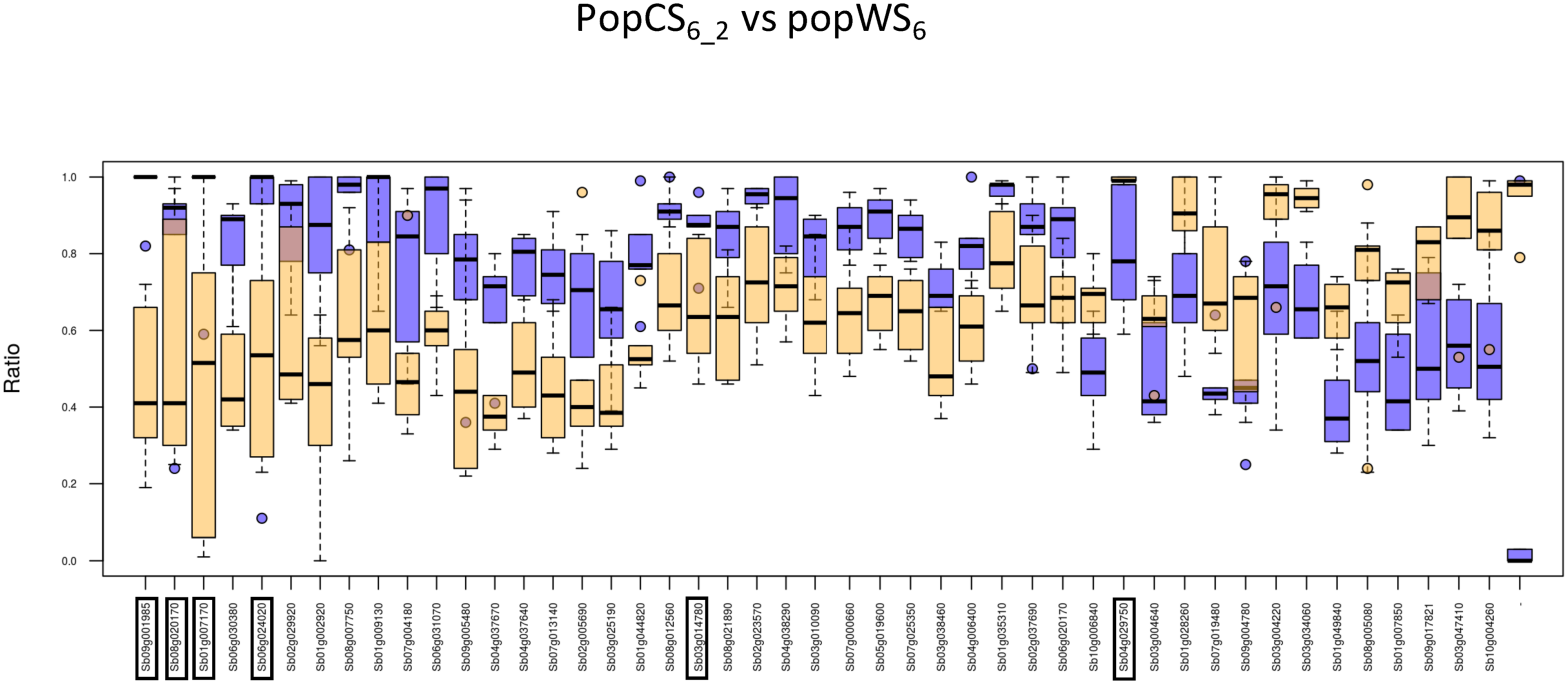
Genes with a contrasted e_T_-ratio median in cultivated (popCS_6_2_) and wild (popWS_6_) samples. Box plot representations of the e_T_-ratio in the cultivated (blue) and wild (orange) sample. The framed genes are common to Fig 4.

Finally, we were looking for genes having an e_T_-ratio (isoform expression balance) that strongly differs in wild and cultivated population. More precisely, we were searching for genes with a difference of e_T_-ratio median value in cultivated and wild compartments greater than 0.2. For this filter, we added the constraint that the median difference should also be superior to the average intrapopulation e_T_-ratio spread, leading to the following filter formulation: |med_r_(CS)- med_r_(WS)| > max(0.2, (iq_r_ (CS) + iq_r_ (WS))/2). This provides us with genes that have a difference in e_T_-ratio between the two compartments (cultivated / wild) that exceed differences observed within compartments (Fig 5). A total of 47 genes were identified when comparing popCS_6_2_ and popWS_6_, among which fifteen were common to the three population comparisons we are focusing on, but this time, we found no over represented GO-term among these genes.

Six genes are common to both comparisons and are contrasted between the cultivated and wild compartments for both e_T_-ratio interquartile and median: Sb01g007170, Sb08g020170, Sb03g014780, Sb06g024020, Sb09g001985 and Sb04g029750. These genes are framed in Figs 4 and 5.

We then tried to estimate the potential impact of the AS events for those genes, by comparing the ‘alternative’ protein to the canonic one predicted according to the annotation of each gene in the sorghum genome version Sbi1.4 (cf. M&M section). The AS events were classified into 4 categories. In the first category, the start codon of the canonic form is not present anymore (no translation prediction is made). In the second category, a stop codon appeared very early (in the first 20% of canonical protein), often due to an early frame shift. In these two cases we can speculate that the AS event may be deleterious (although it can have a role in mRNA degradation). In the third category, the alternative protein is slightly different from the canonical one (*i*.*e*. either with indels affecting less than ten percent of the protein, or identical on more than 50% of the protein but with an equivalent length, or with an identical sequence on a minimum length of 500 amino acids). In the last category the two proteins are 100% identical (*i*.*e*. the AS event concerned only UTR). We can speculate that the alternative protein isoform is functional in these two last categories and that the equilibrium between both mRNA isoforms may have a biological significance (either regulation of amounts of protein, or different roles of the protein themselves). Table 5 provides the distribution, in the 4 above mentioned categories, of the genes having a contrasted e_T_-ratio interquartile in cultivated and wild compartments (genes detailed in Fig 4).

**Table 5:** Distribution of genes with contrasted e_T_-ratio interquartile in cultivated and wild compartments (Fig 4) according to the potential functional impact of the alternative isoform.

Genes with larger e_T_-ratio interquartile in	Identical protein	Potentially functional protein	Total ‘functional’	‘Non functional’ protein	No protein identified	Total ‘deleterious’
Cultivated (Fig 4 left)	8	10	**18 (95%)**	0	1	**1 (5%)**
Wild (Fig 4 right)	10	10	**20 (53%)**	2	16	**18 (47%)**

Finally, in order to go further with the interpretation of our results, we were trying to determine the function of the genes identified as having a contrasted e_T_-ratio distribution, and, in particular, to look for genes potentially involved in traits related to the domestication syndrome.

As mentioned above, Sb03g014780 belongs to the GO term ‘regulation of biological quality’ which was over-represented in the gene-set presenting the highest e_T_-ratio interquartile contrast (Fig 4). This gene is also identified as having a high e_T_-ratio median difference between the wild and the cultivated compartment (Fig 5, framed). The protein predicted for this gene presents 96% of identity with the protein accession Q7G8Y3.2 encoded by the rice gene Os01g0367900. This protein corresponds to a probable chromatin-remodeling complex ATPase chain also known as ISW2 (Imitation Switch Protein 2) which is involved in coordinating transcriptional repression in saccharomyces cerevisiae [56]. The alternative isoform is lacking 28 amino acids, located in a region where three nucleotide binding sites are detected. The deletion is located precisely between the two last nucleotide binding sites, resulting in the merging of those sites. Consequently, in the alternative protein isoform only two nucleotide binding sites are detected. Experimental data would be needed to investigate if its efficiency is affected as it can be predicted from the in silico analysis.

Two other genes annotated by the above-mentioned GO term ‘regulation of biological quality’ and having contrasted e_T_-ratio interquartile, are homologous to genes identified in selection scan studies or genome wide association studies (GWAS) in other species, underlying their putative impact on plant phenotype.

The first one is Sb02g025740. The protein predicted from this gene presents 69% identity with the protein accession Q8LCQ4.1 which is encoded by the LHCA6_ARATH locus from *Arabidopis thaliana* (At1g19150). This protein corresponds to a Photosystem I light harvesting chlorophyll a/b also known as Light Harvesting Complex. These proteins, through their interactions with the core complexes of both photosystems, are involved in the enhancement and regulation of light-harvesting, the transfer of light energy to the photosynthetic reaction centers and also provide protection against photo-oxidative stress [57]. Photosystem Light harvesting chlorophyll a/b proteins have been identified through genome wide association studies as being involved in the photochemical reflectance index in Soybean [58] and to several agronomical traits (height, spike length, number of grains per spike, thousand grain weight, flag leaf area and leaf color) in barley [59]. The alternative splicing event identified for this gene is showing an insertion of only one amino acid in position 16, the rest of the protein is 100% identical to the canonic form.

The second gene is Sb10g025250. Its derived protein presents 73% identity with the protein accession Q949Y3 encoded by At5g34850. This protein corresponds to a bifunctional purple acid phosphatase. Purple acid phosphatases are known to be involved in phosphate acquisition and play a role in phosphate deficiency adaptations [60, 61]. In a recent study on soybean, the gene *GmACP1* was identified as playing a significant role in soybean tolerance to low phosphorus [62]. In addition, in *Helianthus annuus*, evidence of selective sweeps combined with higher than expected Fst values were also identified for a purple acid phosphatase [63].

The last gene identified with a high e_T_-ratio interquartile difference (Fig 4) and for which a function can be predicted is Sb04g030590. This gene codes for a protein showing 93% identity with a soluble inorganic pyrophosphatase (Q0DYB1) encoded by the rice gene Os02g0704900. This protein catalyzes the irreversible hydrolysis of pyrophosphate [64]. In apple, one locus showing signature of selection between wild and domesticated apples was located in a gene coding for an inorganic pyrophosphatase, and this function is described as associated with sugar metabolism and acidity [65]. Indeed, Fruit quality traits have played critical roles in domestication of the apple [65].

Finally, among genes for which a high difference of e_T_-ratio median value is observed between the wild and cultivated sample (Fig 5), the gene Sb1g007850 is potentially involved in ‘the flowering pathway’, another trait of agronomic interest which is often mentioned as a target of the domestication process. Indeed this gene presents more than 90% of amino acid identity with the photoreceptor phytochromes C, from several grass species including rice, maize and *Brachypodium dystachion*. In the temperate model grass *Brachypodium dystachion*, phytochromes C has been shown to be an essential light receptor involved in photoperiodic flowering [66]. In pearl millet, natural variations at the phytochrome C locus are linked to flowering time and morphological variations [67]. The alternative isoform detected with our RNAseq data does not comprise the start codon of the canonical form. Only one copy of phytochrome C is identified in sorghum and it is tempting to speculate that the alternative isoform may be deleterious. The e_T_-ratio between both forms is clearly different between the wild and the cultivated compartment (Fig 5). However, drawing conclusions about a potential selective effect at this locus, linked to domestication would require additional investigations.

## Discussion

Domestication has been shown to impact phenotypic traits, genetic diversity, and gene expression and to be associated with selective effects on a wide number of loci. One study comparing AS profiles in domesticated maize and its wild relative teosinte, has recently been published [39]. To our knowledge, this is the sole publication comparing AS between wild and cultivated plants, and nothing at all has been published so far regarding the impact of domestication on the relative expression of gene isoforms or, more generally, on the diversity of AS expression levels. Here we relied on available RNAseq data to document AS expression variability between wild and cultivated sorghum.

### Strict filters are needed to focus on ‘non-erratic’ AS events

The biological meaning of the complex splicing landscape is still not totally understood. Within the population of mRNA molecules, some variants are issued from random splicing errors and can be assimilated to background noise. Those erratic AS events are not supposed to be present in high frequency. They can therefore be eliminated, or at least strongly minimized, by increasing the sequencing coverage threshold used to assert the presence of isoforms. The remaining AS events may be qualified as ‘non-erratic’ AS events and may have a positive, neutral or negative impact on the organism. They are, somehow, controlled and induced by genetic and/or environmental factors and should be, at least partially, heritable. As such, they are expected to be consistently found in a given genotype, and potentially in other genotypes of the same species, provided that sequencing and environmental conditions are similar.

Our analyses rely on a subset of genes expressing exactly two isoforms. We applied several filters to remove as many as possible of the erratic AS isoforms *i*.*e*. a minimum coverage (depth of sequencing) over the whole transcript and isoform presence in at least two individuals (one cultivated and one wild). According to the high level of expression of the genes we selected in our dataset, and the observed consistency among wild and cultivated compartments, we are quite confident that the AS events we were focusing on are not background noise of splicing machinery.

### The domestication bottleneck is most likely the cause of the global reduction of e_T_-ratio variability in domesticated sorghum

A strong and significant loss of variability of e_T_-ratio (*i*.*e*. balance of the two isoforms resulting from AS) is observed between wild and cultivated compartments (Tables 3 and 4, Fig 2). This result is observed irrespective of the accession samplings considered for each compartment and thus extremely reliable. If domestication has been shown to substantially reduce nucleotide diversity in a vast range of species, including sorghum [45, 49], we show here, for the first time, that domestication also impacts the regulation of the alternative splicing process itself.

AS regulation appears extremely complex and sensitive to environmental stimuli [24]. In this study, the mRNA extraction conditions were—as much as possible—homogenous for all genotypes, we assume that the variability observed is mainly reflecting the genotypic variability. A parallel can be drawn between our results and the results obtained in beans for which a very clear decrease of gene expression variability (18%) was also detected in domesticated beans as compared to their wild counterparts [36]. This loss of expression variability was interpreted as a direct consequence of the strong loss of genetic diversity observed during common bean domestication (almost 50% in coding sequences) affecting DNA regions involved in transcription regulation. Here we assume that the significant loss in AS variability we observe in sorghum is also due to nucleotide variability loss during domestication, in particular in regions where both *trans* and *cis* elements controlling AS are located. Two additional elements reinforce this hypothesis. First, in maize, the diversity of *cis* regulatory elements has been shown to be reduced by domestication and the *cis* element themselves have been suggested to be targets of selection during domestication [68]. Second, in humans, several studies show that AS is, at least partially, controlled by nucleotide diversity present in genomic regions which are more or less close to the target gene [69, 70, 71]. A reduction of nucleotide variability in these regions, whatever their exact distance to the targeted genes, is expected to impact AS variability. Genome wide association mapping on AS variability using either wild or domesticated plants could help to further document these interactions.

The global loss of e_T_-ratio variability, observed between the cultivated and the wild sorghum compartments, is most likely due to the loss of nucleotide diversity induced by the strong demographic bottleneck caused by domestication. Under this neutral, genetic drift related, assumption, the balance between isoforms is expected to have a minute effect for most genes. However, the cumulative effect over the genome might be an important component of the genetic load incurred by domestication. Results from Table 5 tend to confirm this hypothesis.

Indeed, AS events are approximately equally distributed between ‘functional’ and ‘deleterious’ in genes for which the e_T_-ratio interquartile is higher in the wild compartment, in agreement with the neutral hypothesis of this expression diversity reduction. Note that, though the global trend of AS annotation provided in Table 5 may be informative, each individual AS annotation should not be taken for granted. The assignation of a specific AS event as leading to either a functional or non-functional protein needs to be empirically confirmed. Indeed, although an early stop codon is a gage of loss of protein functionality, the effect of the other mutations is not as easily predictable.

### The e_T_-ratios may provide valuable insight for a better understanding of the domestication syndrome

The extent of the nucleotide diversity loss due to domestication is used to characterize the strength of the demographic bottleneck occurring during the domestication process itself [30]. In sorghum, the strength of the bottleneck has been documented to be around 25% (θ_π_) and 38% (θ_W_) at the whole genome level [49]. When only genic regions are considered the strength of the bottleneck is estimated around 39% (θ_π_) and 34% (θ_W_) [49]. The slopes of the linear regressions between wild and cultivated e_T_-ratio are between 0.52 and 0.63 for e_T_-ratio variance and between 0.47 and 0.60 for e_T_-ratio inter-quartile range (Tables 3 and 4). These values could be seen as another insight of the intensity of the bottleneck but are much higher than those derived from nucleotide polymorphism studies. Although it is hazardous to compare these values (different methods and slightly different datasets) we can conclude that the impact of domestication on AS is strong. It is also possible that the nucleotide diversity reduction impacted some loci with pleiotropic effects on AS regulation. The impacts of domestication on AS would deserve to be explored in other species in order to determine whether such a large impact is specific to sorghum or if it is a general trend among domesticated species.

After having discussed the fact that the global decrease of e_T_-ratio variability in the domesticated compartment can be interpreted as a consequence of demographic bottlenecks, we now ask whether this result is entirely neutral (affected by demographic events only), or if it could also result from selective effects. In other words, may a given isoform, or ratio between two isoforms, have increased in frequency in the cultivated compartment because it procures an advantage in the domesticated context, as found for key genes controlling the domestication syndrome [30, 31]. At the genome scale, domestication tends to reduce diversity, however, a gain of diversity can be locally associated with the post domestication diversification especially for loci responsible for interesting traits. This possibility is supported by the results provided in Table 5. Indeed, most AS events identified in genes for which the e_T_-ratio interquartile is higher in cultivated compartments corresponds to potentially ‘functional’ events (whereas AS events found in genes where e_T_-ratio interquartile is lower in cultivated compartments are almost equally distributed between functional and non-functional).

To further confirm this hypothesis we looked closer at the genes showing the most extreme changes in e_T_-ratio median value or interquartile. We found that the ‘regulation of biological quality’ GO annotation was over-represented among the genes for which the e_T_-ratio interquartile in cultivated vs wild sorghum differs the most. Most genes showing the strongest AS e_T_-ratio differences (outliers) are highly homologous to genes of other species shown to be involved in the genetic control of phenotypic traits related to the domestication syndrome. It could be worth to conduct a deeper functional analysis of the few remaining unannotated outlier genes. We are convinced that such AS e_T_-ratio signatures could reveal domestication genes otherwise missed by more traditional methods of selection footprint detection or quantitative genetic approaches (QTL/GWAS).

Finally, in the same way that nucleotide diversity is a mutation reservoir on which natural selection acts, AS can be seen as a leverage on which selection may act too. It should also be kept in mind that AS is a mechanism which can be mobilized to respond to environmental stresses (recently reviewed in [23, 24, 25]). The loss of AS variability caused by domestication is contributing to the domestication load, and probably affects the adaptability potential of crops. This result also underlines the key importance of the conservation and management of the wild compartment to ensure its mobilization in the breeding process of cultivated genotypes.

## Supporting information

S1 Table Isoforms list. In this table are listed, for each isoform, 1) the locus (‘gene_id’), 2) the cufflink_id, 3) the origin of the isoform (described in the publically available annotation or new identified isoform by ‘cufflink’), 4) the predicted protein (when possible), 5) the length of the protein and 6) the identifier of the mRNA under which the sequence is named in the fasta file (S1 File). Note that for some alternative isoforms the start codon of the canonical isoform (given in the publically available annotation) is not present anymore in the alternative mRNA, making protein prediction hazardous, and is then noted ‘start_codon_not_found’.
(XLSX)

S1 File Fasta file containing the mRNA sequences of the 2794 isoforms. (FASTA)
